# Analytical Problems in Separation of Selenomethionine and Its Oxidative Product in HILIC HPLC

**DOI:** 10.3390/molecules26165073

**Published:** 2021-08-21

**Authors:** Aleksandra Sentkowska, Krystyna Pyrzynska

**Affiliations:** 1 Heavy Ion Laboratory, University of Warsaw, 02-093 Warsaw, Poland; 2 Department of Chemistry, University of Warsaw, 02-093 Warsaw, Poland; kryspyrz@chem.uw.edu.pl

**Keywords:** selenomethionine, oxidation, green tea, HILIC-HPLC

## Abstract

Selenomethionine (SeMet) is one of the main selenium forms in foods and supplements. Determining its presence in natural food samples creates difficulties due to possible oxidation processes. The objective of this study was to evaluate the possible degradation of SeMet in water extracts of green teas, one of the most consumed beverages worldwide. Such a medium has not been investigated at this time. The HILIC-HPLC MS/MS method with different stationary phases was used to achieve the satisfactory separation of SeMet and selenomethionine oxide (SeMetO). The addition of dithiothreitol and β-mercaptoethanol, recommended to ensure that SeMet is kept in the reduced form, was also evaluated. The best separation was achieved using the zwitterionic HILIC stationary phase coupled to mass spectrometry and MeOH with water (85/15, *v*/*v*) as the eluent. Extraction was done with hot water with the addition of β-mercaptoethanol. The infusions prepared from Lung-Ching teas (from the Zhejiang Province in China) contained the highest concentration of selenium in a typical cup of tea (12.5–17.3 µg L^−1^). For other tested teas it decreased in the following order: Yunnan > Dilmah > Lipton. For Lung-Ching teas, the sum of concentrations of SeMet and SeMetO corresponded to about 46–63% of the total selenium in their extracts.

## 1. Introduction

Selenium has been recognized as an essential nutrient for humans due to its role in antioxidant selenoproteins, which protect against oxidative stress, maintain intracellular redox status and take part in thyroid hormone production [1,2,3]. Diet is a major source of selenium. Its intake depends on the kind and amount of consumed food, as well as selenium content in food products. However, dietary sources of selenium vary from country to country [4,5]. As some regions around the world are naturally deficient in soil selenium, designing Se functional foods and nutritional supplements [6,7,8,9] or the use of Se-enriched fertilizers during crop cultivation [10,11] has been recommended.

The biological effect and bioavailability of selenium depend not only on the total ingested amount, but also on its chemical form, accessibility and the presence of other dietary components [12,13]. Most plants can absorb inorganic Se and transform it into organic species, which are less toxic and more bioavailable than inorganic forms. The major selenium-containing amino acids occurring naturally in dietary sources are selenomethionine (SeMet) and Se-methylselenocysteine (MeSeCys) [14]. Both display strong antioxidant activities and cytotoxicity against several cancer cells [15]. Detailed knowledge on individual selenium species is important to their potential for human health and to understand the metabolic processes in plants.

The SeMet oxidation process, which takes place during the extraction or storage of the sample, can be observed by the appearance of an additional signal on the chromatogram, with a simultaneous decrease in the intensity of the signal from SeMet [16,17,18,19,20,21,22,23,24]. It was postulated that selenomethionine oxide (SeMetO) and methyloselenone are the possible reaction products. Additives such as dithiothreitol (DTT) or β-mercaptoethanol (βME) are recommended to ensure that SeMet is kept in a reduced form. It has been shown that oxidation of SeMet was favored at lower temperatures, which might have implications for storage stability [17,23]. It is also postulated that the oxidation process of SeMet is strictly connected with enzymatic extraction [22]. The use of enzymatic methods requires an enzymatic sample preparation system and is time consuming (a long time is required for hydrolysis to complete). Larsen et al. found that the amount of SeMetO in Se-enriched yeast using protease XIV was significantly higher than when a sequential of β-glucosidase or a protease mixture was used [21]. The process of SeMet oxidation by flavin-containing monooxygenases has also been reported [23]. Moreover, SeMetO can be reduced back in the presence of glutathione and other thiol compounds, which causes the decrease in the concentration of these thiols. On the other hand, Bierla et al. reported that the SeMetO originally present in the sample cannot be converted to SeMet [18].

Liquid chromatography operating in different modes is the most used technique in selenium speciation analysis [25,26,27]. In anion-exchange chromatography the Hamilton PRP-X100 column is typically used, but SeCys_2_ and SeMetO coelute prevented their clear discrimination [19,20,21,28]. Thus, the combination of size-exclusion and ion-exchange mechanisms was proposed [28]. Cation exchange HPLC separation was also used, but the resolution of inorganic selenium species was not sufficient for quantitative analysis [16,22]. In recent years, hydrophilic interaction liquid chromatography (HILIC) is increasingly being adopted by researchers [29,30,31]. The main reason for the popularity of this technique is that HILIC can provide retention and separation of polar compounds, which are not retained onto non-polar stationary phases used in RP-HPLC or are too strongly retained on polar stationary phases used in NP-HPLC. Additionally, samples prepared in organic solvents can be directly injected into HPLC systems without loss of separation efficiency. This can be obtained due to the fact that the solvent strength in the HILIC mode is roughly inverted to what is observed in the RP mode. However, HILIC is also quite popular in the separation of water samples. It was used for simultaneous separation of phenolic compounds as well as monosaccharides from willow bark extract [32]. Filtration of the sample was the only sample preparation step, which makes the method very competitive, for example, in relation to gas chromatography (GC), where it is necessary to derivatize the sample. The best separation was achieved using 30% MeOH and ACN as eluent (20/80, *v*/*v*) and a Phenomenex Luna Omega Sugar column. The authors note that the addition of ACN to the samples was necessary due to the peak splitting, which was observed for phenolic glycosides. The HILIC mode was also used in the chromatographic analysis of selenohomolanthione in torula (*Candida utilis*) yeast [33]. The combination of amide stationary phase with the eluent, consisting of ACN and 10 mM ammonium formate (pH 5.5) in gradient elution, not only enabled the identification and quantification of selenohomolanthione but also showed the presence of a number of other selenocompounds (one of them was identified as Se-adenosyl-L-homocysteine). From the point of view of the described study, highly organic solvents used in the HILIC mode are also crucial. They enhance the ionization efficiency in the ESI ion source, which provides a significant increase in the detection sensitivity when the HILIC mode is used (in comparison to the RP mode) [34]. This is particularly important in the speciation analysis of selenium, where selenium is present in very low concentrations in natural samples.

The objective of this study was to evaluate the possible degradation of selenomethionine in water extracts as such a medium has not been investigated at this time. Green tea samples from different origins were selected because they are highly consumed beverages and exhibit health-promoting properties [35]. Hydrophilic interaction liquid chromatography coupled with a tandem mass spectrometry (HILIC-MS/MS) method with different stationary phases was used to achieve the satisfactory separation. The addition of DTT and βME was also evaluated to avoid the oxidation of SeMet.

## 2. Results and Discussion

### 2.1. Total Se in Tea Leaves and Infusions

Table 1 presents total selenium content in the studied green teas obtained after microwave digestion of their leaves. The green teas from Zhejiang Province (Lung-Ching) showed the highest content of Se, ranging from 4.63 µg g^−1^ to 5.13 µg g^−1^, followed by Yunnan teas. The popular brands from Sri Lanka contained the lowest content of selenium, 3.09 ± 0.09 µg g^−1^ for Dilmah brand and 2.96 ± 0.07 µg g^−1^ for Lipton, respectively. It was reported that all the varieties of green tea contained Se in the range of 0.017–6.590 μg g^−1^ [8]. Those with a selenium level ≥5.00 μg g^−1^ are considered as high-Se tea, those in the range of 0.35–5.00 μg g^−1^ as medium high and those in the range of 0.10–0.35 μg g^−1^ as medium. Tea leaves containing less than 0.10 μg g^−1^ of Se are classified as low. Thus, green teas from Zhejiang Province can be classified as high-Se teas.

Owing to traditional consumption of green tea infused in hot water, the total content of selenium was determined in water extracts of the studied teas. Hot water, buffered solution or HCl-alcohol mixtures extract only low molecular weight selenium compounds as free non-protein bounded selenoamino acids [36]. The obtained results are presented in Table 1.

The water extracts prepared from 2 g of Lung-Ching green teas in 200 mL of water contained the highest concentration of selenium in a typical cup of tea in the range of 12.5–17.3 µg L^−1^. For other tested tea infusions, Se concentration decreased in the following order: Yunnan > Dilmah > Lipton. The efficiency of selenium extraction decreased in the same direction. The regression analysis between Se content in dried green tea leaves and its concentration in water extracts gave good linear correlation (R^2^ = 0.9914), suggesting that the leaching rate of selenium in tea infusions is closely related to its content.

The level of selenium concentration in the tea infusions may be affected by extraction conditions (amount of material relative to water, infusion time and temperature) as well as organic matrix of corresponding tea and its original mineral content. A total of 23.8% selenium was extracted from Se-rich green tea using hot water, while for Yunnan Lan Xiang tea, it was equal to 15.4% [37]. Chen et al. obtained 17.4% efficiency in selenium extraction from Chinese Se-rich green tea after repeating this process three times [38]. For black, green and oolong teas purchased in local stores in Japan, the percentage of Se extractable from water was generally less than 5%, and for that sold under the name “high Se tea”, it was more than 20%, which confirmed our results [38].

Several institutions have proposed reference values for the recommended dietary allowance (RDA) of selenium. There are variations across Europe and worldwide, but most levels are in the range of 50–60 μg/day [39]. Drinking three cups of Lung-Ching green tea will contribute to about 16% of the daily requirement for selenium.

### 2.2. Speciation Analysis

**Figure 1:**
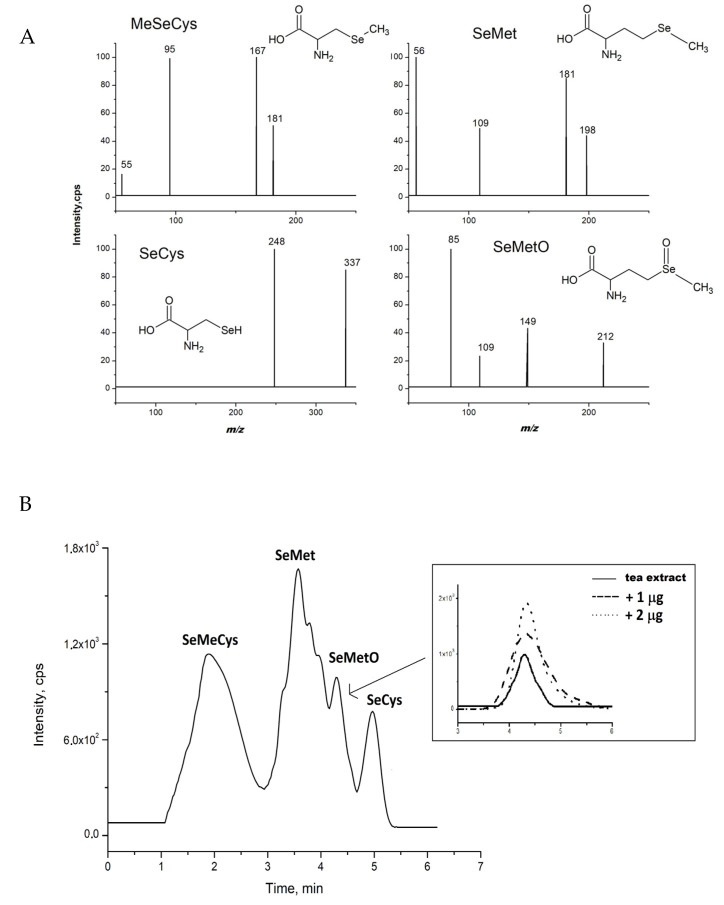
(**A**) The product ion spectra of organic selenium species present in water extract of Lung-Ching tea; (**B**) Chromatogram of green tea water extract (sample Lung-Ching). Atlantis HILIC column, eluent: methanol and water, (85/15, *v*/*v*). Inset SeMetO peaks after addition of its standard solutions.

The chromatogram of water extract of Chinese green tea Lung-Ching (sample 3), under the conditions described earlier [29,40], is presented in Figure 1B. The analytes were identified by comparing retention times and m/z values obtained by MS and MS^2^ mass spectra (Figure 1A). No traces of Se(IV) and Se(VI) were detected (retention times 1.2 and 1.8 min, respectively, limit of detection 0.10 μg L^−1^ [29]). Inorganic selenium compounds were detected in enriched-Se green tea where its leaves were sprayed during cultivation with Na_2_SeO_3_ salt [41]. Inorganic forms of selenium can also be found in other selenium-supplemented plants, not only in tea. About 60% of inorganic Se in the form of Se(IV) was present in the non-supplemented rice, while only 6.8% was found in Se-enriched rice extract [42].

As no inorganic species of selenium were detected in the extracts, all analysis focused on its organic forms. Selenomethionine was the major species in all analyzed green tea extracts. However, conversion of SeMet to selenomethionine oxide was observed in the extracts as a separate peak at a retention time of 4.35 min. SeMetO was fragmented at 21 eV, and collision energy applied in the MS/MS spectrum showed more prominent fragments at *m*/*z* of 85 (mass spectra of organic selenium species are presented in Figure 1A). This phenomenon resulted in difficulties in the quantitative analysis of SeMet and SeMetO. Additionally, the presence of SeMetO was confirmed by the addition of its standard solutions to the studied tea infusion (Figure 1B). It should be mentioned that the signal corresponding to the presence of SeMetO was registered only for Chinese green tea Lung-Chin infusions.

Compared to the previously proposed method for selenium speciation analysis [29,40], where SeMetO was not evaluated, the aqueous component of the mobile phase (ammonium acetate pH 7) was replaced with water. This resulted in the change in the elution order—MeSeCys was eluted before SeMet. Buffers are recommended in HILIC to increase the polarity of the water layer adsorbed on the stationary phase [30]. The obtained elution order suggests that the hydrophilic partition of the analytes governs the retention mechanism. On the other hand, at pH 7 selenium compounds are negatively charged (pH above their pKa values), so they can also compete with the buffer ions for a place to interact with the stationary phase. In such cases, the electrostatic interactions also play a significant role in the separation mechanism. When both mechanisms are equally important, changing the eluent composition causes one of them to dominate, which results in changes in the separation pattern.

**Figure 2:**
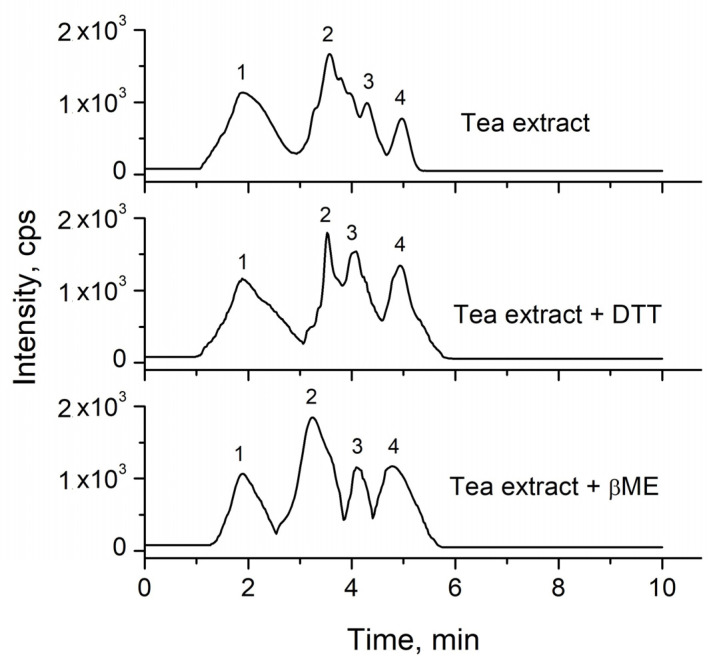
Chromatograms of water extract of green tea (sample Lung-Ching-3) without and with the addition of DTT and βME. Each addition was made 5 min after the addition of water to the tea sample. Atlantis HILIC column, eluent: methanol and water, pH 5.8 (85/15, *v*/*v*). 1: MeSeCys; 2: SeMet; 3: SeMetO; 4: SeCys.

The data in the literature reported, as mentioned earlier, that oxidation of selenomethionine can be prevented in the presence of dithiothreithol (DTT) or β-mercaptoethanol (βME). Thus, the addition of both reducing agents was tested in the extraction procedure of green teas by hot water. The obtained chromatograms are presented in Figure 2.

The addition of DTT or βME did not completely solve the problem. The best separation of SeMet and SeMetO was observed in the presence of β-mercaptoethanol, but not to the baseline. Two different ways of adding the reductant to the sample have been investigated. First, it was added in the extraction solvent (water) to the grounded tea sample and then the extraction was done. In the second procedure, the reductant was added about 5 min after the addition of water to the sample. However, such changes in the analytical methodology had no effect on the concentration of SeMet and SeMetO (data not shown), suggesting that the selenooxide was already present in dried tea. SeMetO occurred as an oxidation product of SeMet even when dissolved oxygen was removed throughout sample preparation and the chromatographic runs [21]. Krata et al., using species-specific isotope dilution HPLC ICP-MS and the addition of spike solution before enzymatic extraction, reported that the only source of SeMetO in certified reference materials of a different matrix (skimmed powder milk, wheat flour and enriched yeast) is oxidized SeMet [22].

**Figure 3:**
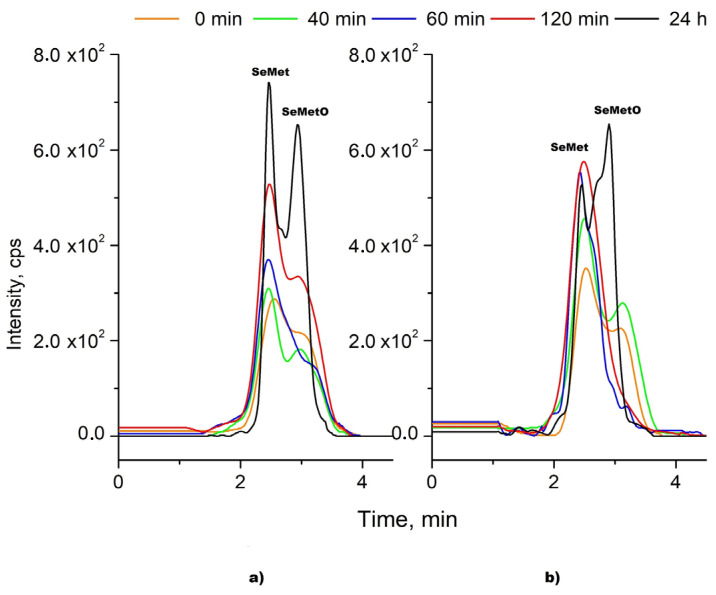
The chromatograms of SeMet and SeMetO mixture standard solutions after the addition of (**a**) βME and (**b**) DTT in the function of time. Column and eluent as in Figure 2.

The kinetic of the reduction process of SeMetO after the addition of both reductants (DTT and βME) was investigated. The chromatograms of SeMet and SeMetO standards mixture were recorded in the function of time after mixing with a given reductant (Figure 3). It was observed that dynamic equilibrium between the reduced and oxidized form of SeMet is established. The SeMetO peak was decreasing and that of SeMet was increasing until 60 min, but up to 24 h later, inverse relationships were observed. Similar results were observed when DTT concentration was increased twice to 10 mM. The best separation was achieved 24 h after βME addition; however, peaks are only half-split.

To our best knowledge, there are no reports regarding the exact mechanism of the reaction between the oxidized form of SeMet and DTT or βME, as well as its kinetic. The conversion of DTT to disulphide, its oxidized form, was observed [43], while Chéry et al. postulated that dithiothreitol can degrade the original amino acids into DTT-Se molecules, probably a cyclic, with the consequent loss of the speciation information [44]. The formed SeMetO (as two diastereomers) can readily undergo facile racemization in aqueous media, mostly via the formation of achiral hydrates [23]. SeMetO can be reduced back to yield SeMet by thiols (glutation, cysteine) and antioxidants such as ascorbic acid [45,46], although SeMetO reduction by thiols was much faster than by ascorbic acid, which needed more excess of reductant [47]. Some authors reported that selenometionine oxide present in selenium-rich yeast, which is a food and feed supplement sold worldwide, cannot be back reduced to SeMet [18]. As can be seen, this issue is more complicated and reported results are sometimes in conflict. Moreover, it should be taken into consideration that matrix components may be involved in these reactions. The reaction may run very differently in the standard solution and in the presence of a sample matrix.

**Figure 4:**
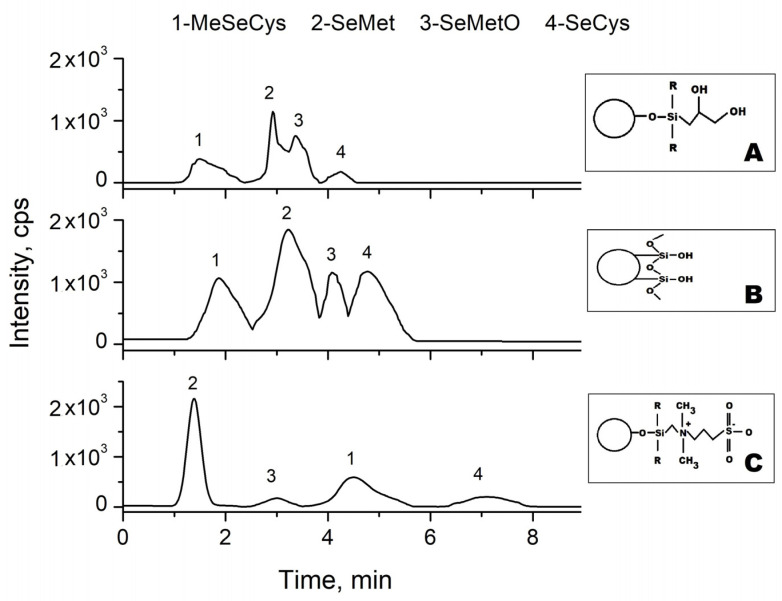
Comparison of the separation efficiency of selenium species in Lung-Ching-1 green tea with the addition of βME on different HILIC stationary phases: (**A**) bare silica, (**B**) diol, (**C**) sulfobetaine (zwitterionic); Mobile phase: 85% MeOH/water in isocratic elution.

In further research, we decided to check the possibility of the use of other HILIC stationary phases to achieve better separation of the signals from SeMet and SeMetO. Their functional groups and obtained chromatograms are presented in Figure 4. In the case of the diol column, the separation was less efficient than that achieved previously onto the silica stationary phase. The elution order obtained on the sulfobetaine stationary phase (ZIC HILIC) was in agreement with the polarity of selenium species, with the exception of SeMetO (logP-2.9) and MeSeCys (logP-2.8); however, the differences in their logP values are rather small. Importantly, the use of the ZIC-HILIC column enabled the complete separation of the SeMet from its oxide. The difference in the retention times of these analytes is about 3 min, and it can be explained by the complex retention mechanism, which takes place on this stationary phase. The ZIC-HILIC column has a zwitterionic stationary phase which contains quaternary ammonium groups and acidic sulfonic groups present in a 1:1 molar ratio. However, a small negative charge near the column surface is observed due to the distance between the silica support and sulfonic group, which is pH dependent [48]. The pKa values of selenium species are in the range from 2.8 to 4.8, so at pH 5.8 (value refers to pH of water component of the eluent), they are all negatively charged. It should be highlighted that in the separation of selenium species, methanol seems to be the better choice as the mobile phase component [29,40]. Methanol is a stronger eluent than acetonitrile and is known to be adsorbed near the stationary phase via hydrogen bonding with the residual silanols, while acetonitrile can react with silanols via dipol–dipol interaction [47]. The best separation of selenium compounds can be achieved using a ZIC-HILIC column. However, the intensity of the signals is lower in comparison to the diol and silica stationary phases.

**Figure 5:**
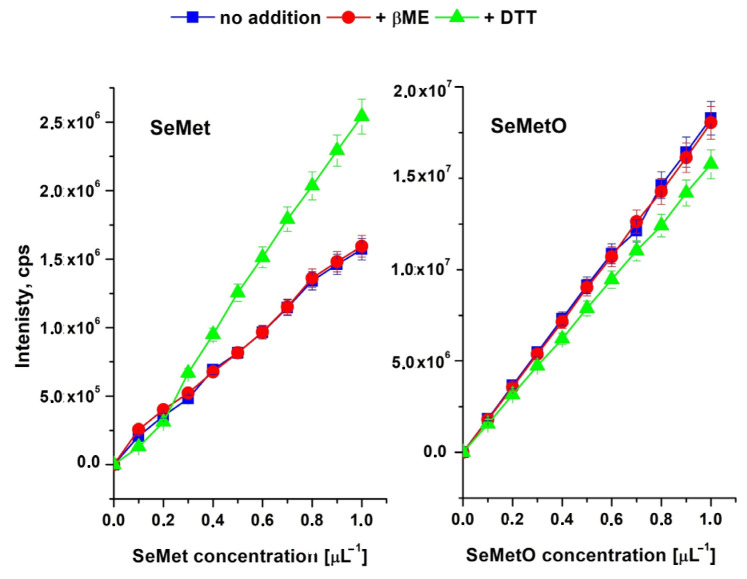
The influence of the DTT (5mM) and βME (0.005%) addition on the slope of the calibration curves for SeMet and SeMetO. ZIC HILIC column, mobile phase: MeOH/ water (85/15, *v*/*v*).

It was found that the addition of DTT affects the slope of the calibration curves for SeMet and SeMetO (Figure 5). The presence of βME standard solutions (0.005%) had no effect on the shape of the curves in comparison to its absence. Thus, in the case of this study, the compromise between the separation of SeMet and SeMetO and the chromatographic conditions should be achieved. The use of zwitterionic stationary phase coupled to mass spectrometry and the eluent consisting of MeOH and water (85/15, *v*/*v*) was considered to be the best. Extraction was done with hot water with the addition of β-mercaptoethanol. The limit of detection (LOD) for the selenium compounds was calculated from its standards and expressed as three times the standard deviation (*n* = 6). LOD was 0.05 µg/L for SeMet, SeMetO and MeSeCys; 0.06 µg/L for Se (VI); and 0.1 µg/L for Se(VI) and SeCys. The LOQ values, expressed as ten times the standard deviation, were as follows: 0.1 µg/L for SeMet, SeMetO and MeSeCys; 0.15 µg/L for Se (VI); and 0.3 µg/L for Se(VI) and SeCys. Table 2 lists the parameters of calibration curves as well as the validation parameters for all studied selenium species. The linearity range of the method was 0.1–1500 µg/L. The repeatability between four series of measurements (six in each) made for the same sample based on the RSD value was 2.2%.

**Figure 6:**
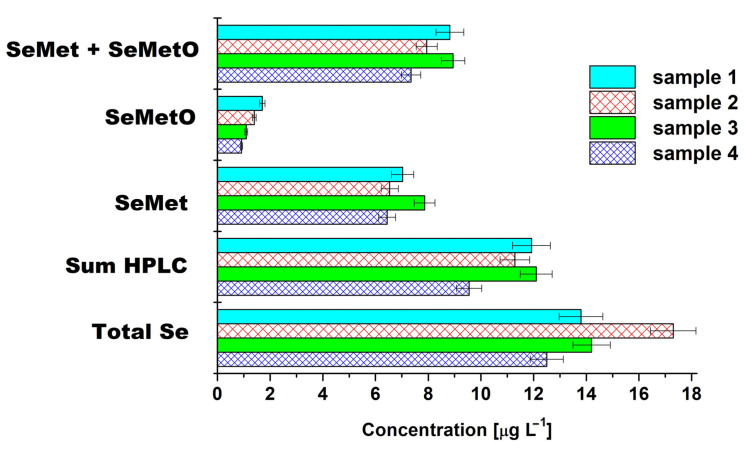
Concentration balance of selenium and its species determined in Lung-Ching green tea samples by ZIC HILIC HPLC separation procedure. Sum HPLC means the sum of the concentrations of SeMet and SeMetO obtained from the chromatographic analysis.

Figure 6 shows the concentration balance for four different samples of infusions of Lung-Ching green tea. The sum of chromatographically determined concentrations of all organic selenium species (marked as Sum HPLC) amounted to 66–86% of the total content of selenium in the extracts, whereas the sum of concentrations of SeMet and SeMetO corresponded to 46–63% of the total Se. It should be noticed that the highest concentration of total Se in the water extracts was determined in sample 2 (17.3 ± 0.123 µg L^−1^). The lowest percentages in the above dependencies also correspond to this sample. Although, according to the producers, all the studied green tea samples (named Lung-Ching) come from Zhejiang Province, a seleniferous region in southern China, the content of individual selenium species was different between them. Analysis of variance and significant differences among means were performed with one-way ANOVA. Mean values of SeMetO concentration were significantly different in Tukey’s test (*p* ≤ 0.05) for all pairs of Chinese green tea extracts, while for concentration of SeMet, these significant differences were observed for two pairs (sample 1 and 2, sample 2 and 3). These tea samples were collected from different shops. Thus, it is possible that differences in SeMeO concentration may be connected with the aging of tea leaves or their storage conditions.

## 3. Materials and Methods

The commercial standards of sodium selenite (Na_2_SeO_3_), sodium selenate (Na_2_SeO_4_), selenomethionine (SeMet), Se-methylselenocysteine (MeSeCys) as well as the other chemicals (including dithiothreitol (DTT) and β-mercaptoethanol (βME) were purchased from Merck-Sigma (Steinheim, Germany). Selenomethionine oxide (SeMetO) was achieved by the addition of 1 mL of 30% (*v*/*v*) H_2_O_2_ to 10 mL of a 0.1 mol L^−1^ HCl solution with selenomethionine (1 mg Se L^−1^) and left overnight in the dark [19]. Methanol was of HPLC grade from Merck (Darmstadt, Germany). Ultrapure water from Milli-Q system (Millipore, Bedford, MA, USA) was used in all experiments.

The analyzed tea samples were purchased in Warsaw tea shops. Chinese teas include 4 selenized green teas Lung-Ching from Zhejiang Province and 2 green teas from Yunnan Province as well as other popular teas from Sri Lanka (Dilmah produced by Dilmah, Colombo, Sri Lanka and Lipton produced by Unilevel, England).

The total selenium content was determined after microwave digestion. A total 4 mL of concentrated HNO_3_ and 1 mL of HClO_4_ were added to 1 g of ground sample and heated with a microwave system (Whirlpool, Comerio, Italy) for 15 min by increasing the microwave power to 160 W and the temperature to 180 °C in a stepwise fashion. The digested solution was then gently heated to remove the excess of HNO_3_ and after cooling transferred to volumetric flasks. The reference material tea leaves (INCT-TL-1) with selenium content of 0.076 µg g^−1^ (from Institute of Nuclear Chemistry and Technology, Warsaw, Poland) was used as a quality control standard to validate the applied procedure. The determined value of (0.071 ± 0.004) µg g^−1^ was in good accordance with the nominal value equals to 0.076 µg g^−1^, showing good accuracy.

The infusions were prepared in order to simulate the household brewing process. 2.00 g of dried tea leaves (typical tea bag) was mixed with 200 mL of hot distilled water (95 °C) for 1 h. Before the analysis, all the extracts were filtered through 0.22 µm PTFE filter (Millipore, Merck, MA, USA). For the investigation of the progress of SeMet oxidation reaction, the solution also contained 5 mM DTT and 10 mM DTT or 0.005% of βME.

Chromatographic analysis was performed with the Shimadzu LC system and consisted of binary pumps LC20-AD, degasser DGU-20A5, column oven CTO-20AC, autosampler SIL-20AC and 8030 triple quadrupole Mass Spectrometer (Shimadzu, Japan) equipped with an ESI source operated in negative-ion or in positive mode, according to the determined species. The ESI conditions were as follows: the capillary voltage 4.5 kV, temperature 400 °C, the source gas flow 3 L min^−1^, drying gas flow 10 L min^−1^.

For separation of selenium species, different columns were used: Atlantis HILIC (100 × 2.1, 3 µm) from Waters, ZIC-HILIC column (100 × 2.1, 3 µm) from Merck and Luna HILIC (100 × 2.0, 3.5 µm) from Phenomenex. The mobile phase consisted of methanol and 8 mmol L^−1^ ammonium acetate pH 7 (85/15, *v*/*v*) or water. The mobile phase was delivered at 0.2 mL min^−1^. Selenium compounds were identified by comparing their retention times and m/z values obtained by MS and MS^2^ with the mass spectra.

## 4. Conclusions

There is an increasing demand for natural products containing selenium due to knowledge about its importance in the human body. However, Se speciation analysis in different matrices, which is needed to define their biological roles, presents a great challenge. The HILIC-MS/MS method, with different stationary phases, was evaluated to achieve the satisfactory separation of selenoamino acids in green tea water infusions. The main problem was the degradation of SeMet and a separation of its peak from selenomethionine oxide, the formed oxidation product. The impact of the selenooxide on the quantitative analysis of selenomethionine was not described. However, it seems to be reasonable in monitoring the changes over time in Se speciation upon storage.

The recommended addition of thiols (DTT and βME) did not completely solve this problem, although, as it was reported, in their presence, the signal from SeMetO should disappear. In our study, upon addition of these reductants, the obtained chromatographic profiles were complicated by broadening and double peaking. Among the tested HILIC columns, the best results were obtained by using a zwitterionic column; however, the intensity of the signals was lower in comparison to the diol and silica stationary phases.

## Figures and Tables

**Table 1:** Selenium in green tea samples and their infusions.

Tea Samples	Dry Leaves (µg g^−1^) *	Infusion (µg L^−1^)	Extraction (%)
Lung-Ching −1	4.63 ± 0.05	13.8 ± 0.110	29.8
Lung-Ching −2	5.13 ± 0.03	17.3 ± 0.123	33.7
Lung-Ching −3	4.73 ± 0.04	14.2 ± 0.100	30.0
Lung-Ching −4	4.60 ± 0.05	12.5 ± 0.100	27.2
Yunnan −1	3.21 ± 0.02	6.83 ± 0.105	21.3
Yunnan −2	3.36 ± 0.08	7.31 ± 0.011	21.8
Dilmah	3.09 ± 0.09	5.63 ± 0.003	18.2
Lipton	2.96 ± 0.07	4.37 ± 0.020	14.8

* Mass of the dry tea leaves used for extraction.

**Table 2:** Parameters of the calibration equations and validation parameters in ZIC-HILIC separation using methanol/water eluent (85/15, *v*/*v*).

Analyte Abbreviation	Slope	R^2^	LOD [µg/L]	LOQ [µg/L]	RSD %
SeMet	1,636,351	0.9999	0.05	0.10	2.2
SeMetO	1,473,217	0.9999	0.05	0.10	2.0
MeSeCys	1,223,582	0.9999	0.05	0.10	2.2
SeCys	18,224	0.9998	0.06	0.30	2.1
Se(IV)	11,304	0.9989	0.06	0.30	2.3
Se(VI)	59,310	0.9997	0.06	0.15	2.4

## Data Availability

The data presented in this study are available on request from the corresponding author.

## Sample Availability

Samples of the compounds are not available from the authors.

## References

[1] Yang R., Liu Y., Zhou Z. (2017). Selenium and Selenoproteins, from Structure, Function to Food Resource and Nutrition. Food Sci. Technol. Res..

[2] Rayman M.P. (2020). Selenium intake, status, and health: A complex relationship. Hormones.

[3] Kuršvietiené L., Mongirdiené A., Bernatoniené J., Šulinskiene J., Stanevičiené I. (2020). Selenium anticancer properties and impact on cellular redox status. Antioxidants.

[4] Pyrzynska K. (2014). Edible plants enriched with selenium. J. Agric. Sci. Technol..

[5] Stoffaneller R., Morse N.L. (2015). A Review of Dietary Selenium Intake and Selenium Status in Europe and the Middle East. Nutrients.

[6] Adadi P., Barakova N.V., Muravyov K.Y., Krivoshapkina E.F. (2019). Designing selenium functional foods and beverages: A review. Food Res. Int..

[7] Zhang X., He H., Xiang J., Li B., Zhao M., Hou T. (2021). Selenium-containing soybean antioxidant peptides: Preparation and comprehensive comparison of different selenium supplements. Food Chem..

[8] Liang J., Puligundla P., Ko S., Wan X.C. (2014). A review on selenium-enriched green tea: Fortification methods, Biological activties and application prospect. Sains Malays..

[9] Constantinescu-Aruxandei D., Frîncu R.M., Capră L., Oancea F. (2018). Selenium Analysis and Speciation in Dietary Supplements Based on Next-Generation Selenium Ingredients. Nutrients.

[10] Wan J., Zhang M., Adhikari B. (2018). Advances in selenium-enriched foods: From the farm to the fork. Trends Food Sci. Technol..

[11] Sarwar N., Akhtar M., Kamran M.A., Imran M., Riaz M.A., Kamran K., Hussain S. (2020). Selenium biofortification in food crops: Key mechanisms and future perspectives. J. Food Compos. Anal..

[12] Moreda-Piňeiro J., Moreda-Piňeiro A., Bermejo-Barrera P. (2017). In vivo and in vitro testing for selenium and selenium com-pounds bioavailability assessment in foodstuff. Crit. Rev. Food Sci. Nutr..

[13] Pyrzynska K., Sentkowska A. (2020). Selenium in plant foods: Speciation analysis, bioavailability, and factors affecting composition. Crit. Rev. Food Sci. Nutr..

[14] Sentkowska A., Pyrzyńska K. (2019). Investigation of antioxidant activity of selenium compounds and their mixtures with tea polyphenols. Mol. Biol. Rep..

[15] Chuai H., Zhang S.-Q., Bai H., Li J., Wang Y., Sun J., Wen E., Zhang J., Xin M. (2021). Small molecule selenium-containing compounds: Recent development and therapeutic applications. Eur. J. Med. Chem..

[16] Kápolna E., Fodor P. (2007). Bioavailability of selenium from selenium-enriched green onions (*Allium fistulosum*) and chives (*Allium schoenoprasum*) after “in vitro” gastrointestinal digestion. Int. J. Food Sci. Nutr..

[17] Liu Q., Bei Y. (2010). Thermodynamics and dynamic kinetics of the oxidation of selenomethionine to methionine selenooxide: A DFT study. Prog. React. Kin. Mech..

[18] Bierla K., Szpunar J., Yiannikouris A., Łobiński R. (2012). Comprehensive speciation of selenium in selenium-rich yeast. TrAC Trends Anal. Chem..

[19] Michalska-Kacymirow M., Kurek E., Smolis A., Wierzbicka M., Bulska E. (2014). Biological and chemical investigation of *Allium cepa* L. response to selenium inorganic compounds. Anal. Bioanal. Chem..

[20] Moreda-Piñeiro J., Sánchez-Piñero J., Mañana-López A., Turnes-Carou I., Alonso-Rodríguez E., López-Mahía P., Muniategui S. (2018). Selenium species determination in foods harvested in Seleniferous soils by HPLC-ICP-MS after enzymatic hydrolysis assisted by pressurization and microwave energy. Food Res. Int..

[21] Larsen E.H., Sloth J., Hansen M., Moesgaard S. (2003). Selenium speciation and isotope composition in 77SeSe-enriched yeast using gradient elution HPLC and ICP-dynamic cell-MS. J. Anal. At. Spectrom..

[22] Krata A.A., Wojciechowski M., Karasinski J., Bulska E. (2018). Comparative study of high performance liquid chromatography species-specific and species-unspecific isotope dilution inductively coupled plasma mass spectrometry. A case study of selenomethionine and the origin of its oxidized form. Microchem. J..

[23] Krause R.J., Glocke S.C., Sicuri A.R., Ripp S.L., Elfarra A.A. (2006). Oxidative metabolism of seleno-L-methionine to L-methionine selenooxide by flavin-containing mono-oxygenases. Chem. Res. Toxicol..

[24] Pedrero Z., Encinar J.R., Madrid Y., Cámara C., Zayas Z.P. (2007). Application of species-specific isotope dilution analysis to the correction for selenomethionine oxidation in Se-enriched yeast sample extracts during storage. J. Anal. At. Spectrom..

[25] LeBlanc K.L., Kumkrong P., Mercier P.H., Mester Z. (2018). Selenium analysis in waters. Part 2: Speciation methods. Sci. Total Environ..

[26] Pyrzynska K., Sentkowska A. (2019). Liquid chromatographic analysis of selenium species in plant materials. TrAC Trends Anal. Chem..

[27] Bierla K., Godin S., Łobiński R., Szpunar J. (2018). Advances in electrospray mass spectrometry for the selenium speciation: Focus on Se-rich yeast. TrAC Trends Anal. Chem..

[28] Pedrero Z., Encinar J.R., Madrid Y., Cámara C. (2007). Identification of selenium species in selenium-enriched Lens esculenta plants by using two-dimensional liquid chromatography-inductively coupled plasma mass spectrometry and [77Se]selenomethionine selenium oxide spikes. J. Chromatogr. A.

[29] Sentkowska A., Pyrzyńska K. (2018). Hydrophilic interaction liquid chromatography in the speciation analysis of selenium. J. Chromatogr. B.

[30] Buszewski B., Noga S. (2012). Hydrophilic interaction liquid chromatography (HILIC)—A powerful separation technique. Anal. Bioanal. Chem..

[31] Dejaegher B., Mangelings D., Heyden Y.V. (2008). Method development for HILIC assays. J. Sep. Sci..

[32] Meriö-Talvio H., Dou J., Vuorinen T., Pitkänen L. (2021). Fast HILIC Method for Separation and Quantification of Non-Volatile Aromatic Compounds and Monosaccharides from Willow (*Salix* sp.) Bark Extract. Appl. Sci..

[33] Bierła K., Suzuki N., Ogra Y., Szpunar J., Łobiński R. (2017). Identification and determination of selenohomolanthionine–The major selenium compound in Torula yeast. Food Chem..

[34] Sentkowska A., Biesaga M., Pyrzyńska K. (2015). Retention Study of Flavonoids under Different Chromatographic Modes. J. Chromatogr. Sci..

[35] Senanayake S.P.J.N. (2013). Green tea extract: Chemistry, antioxidant properties and food applications—A review. J. Funct. Foods.

[36] Yoshida M., Kimura Y., Abe M., Ando T., Tachi H., Fukunaga K. (2001). Quantitative Evaluation of Selenium Contained in Tea by High Performance Liquid Chromatography. J. Nutr. Sci. Vitaminol..

[37] Wen S., Zhu X., Wei Y., Wu S. (2013). Cloud point extraction-inductively coupled plasma mass spectrometry for separa-tion/analysis of aqueous-exchangeable and unaqueous-exchengeable selenium in tea samples. Food Anal. Methods..

[38] Chen S., Zhu S., Lu D. (2015). Solidified floating organic drop microextraction for speciation of selenium and its distribution in selenium-rich tea leaves and tea infusion by electrothermal vapourisation inductively coupled plasma mass spectrometry. Food Chem..

[39] Hurst R., Collings L., Harvey J., King M., Hooper L., Bouwman J., Gurinovic M., Fairweather-Tait S.J. (2013). EURRECA—Estimating selenium requirements for deriving dietary reference values. Crit. Rev. Food Sci. Nutr..

[40] Sentkowska A. (2021). Content of selenoaminoacids and catechins in Chinese green teas. Eur. Food Res. Technol..

[41] Yu F., Sheng J., Xu J., An X., Hu Q. (2006). Antioxidant activities of crude tea polyphenols, polysaccharides and proteins of selenium-enriched tea and regular green tea. Eur. Food Res. Technol..

[42] Fang Y., Zhang Y., Catron B., Chan Q., Hu Q., Caruso J.A. (2009). Identification of selenium compounds using HPLC-ICPMS and nano-ESI-MS in selenium-enriched rice via foliar application. J. Anal. At. Spectrom..

[43] Obieziurska-Fabisiak M., Pacuła A.J., Capoccia L., Drogosz-Stachowicz J., Janecka A., Santi C., Ścianowski J. (2020). Phenylselanyl group incorporation for “glutathione peroxidase-like” activity modulation. Molecules.

[44] Chéry C.C., Dumont D., Moens L., Vanhaecke F., Cornelis R. (2005). Influence of reducing agents on the integrity of selenocompounds. Exploratory work for selenoproteome analysis. J. Anal. At. Spectrom..

[45] Krause R.J., Elffara A.A. (2009). Reduction of L-methionine selenoxide to seleno-L-methionine by endogenous thiols, ascorbic acid, or methimazole. Biochem. Pharmacol..

[46] Singh B.G., Kumar P., Iwaoka M., Priyadarsini I. (2019). Free radical induced selenoxide formation in isomeric organoselenium compounds: The effect of chemical structures on antioxidant activity. NJC.

[47] Buszewski B., Bocian S., Rychlicki G., Vajda P., Felinger A. (2010). Study of solvent adsorption on chemically bonded stationary phases by microcalorimetry and liquid chromatography. J. Colloid Interface Sci..

[48] Greco G., Letzel T. (2013). Main Interactions and Influences of the Chromatographic Parameters in HILIC Separations. J. Chromatogr. Sci..

